# Vitamin K2 (Menaquinone-7) Reverses Age-Related Structural and Cognitive Deterioration in Naturally Aging Rats

**DOI:** 10.3390/antiox11030514

**Published:** 2022-03-08

**Authors:** Hany A. Elkattawy, Fatma M. Ghoneim, Mohamed Ahmed Eladl, Eman Said, Hasnaa Ali Ebrahim, Mohamed El-Shafey, Saad Mohamed Asseri, Mohamed El-Sherbiny, Reem Hamoud Alsalamah, Nehal M. Elsherbiny, Shimaa Hadhod

**Affiliations:** 1 Department of Basic Medical Sciences, College of Medicine, AlMaarefa University, P.O. Box 71666, Riyadh 11597, Saudi Arabia; hmohammed@mcst.edu.sa (H.A.E.); msharbini@mcst.edu.sa (M.E.-S.); 2 Medical Physiology Department, College of Medicine, Zagazig University, Zagazig 44519, Egypt; dedamodymodydeda@gmail.com; 3 Histology and Cell Biology Department, Faculty of Medicine, Mansoura University, Mansoura 35516, Egypt; fatmaghonaim@gmail.com; 4 Department of Basic Medical Sciences, College of Medicine, University of Sharjah, Sharjah 27272, United Arab Emirates; meladl@sharjah.ac.ae; 5 Department of Pharmacology and Toxicology, Faculty of Pharmacy, Mansoura University, Mansoura 35516, Egypt; 6 Faculty of Pharmacy, New Mansoura University, New Mansoura 7723730, Egypt; 7 Department of Basic Medical Sciences, College of Medicine, Princess Nourah bint Abdulrahman University, P.O. Box 84428, Riyadh 11671, Saudi Arabia; haebrahim@pnu.edu.sa; 8 Department of Anatomy and Embryology, Faculty of Medicine, Mansoura University, Mansoura 35516, Egypt; mmelshafey@fcms.edu.sa; 9 Physiological Sciences Department, Fakeeh College for Medical Sciences, P.O. Box 2537, Jeddah 21461, Saudi Arabia; 10 Department of Clinical Medical Sciences, College of Medicine, AlMaarefa University, P.O. Box 71666, Riyadh 11597, Saudi Arabia; vdean.med@mcst.edu.sa; 11 PharmD Program, Faculty of Pharmacy, University of Tabuk, P.O. Box 741, Tabuk 71491, Saudi Arabia; reemalsalamah1999@gmail.com; 12 Department of Biochemistry, Faculty of Pharmacy, Mansoura University, Mansoura 35516, Egypt; 13 Department of Pharmaceutical Chemistry, Faculty of Pharmacy, University of Tabuk, P.O. Box 741, Tabuk 71491, Saudi Arabia

**Keywords:** aging, Vit. K2, NLRP3, tyrosine, cerebral, hippocampus

## Abstract

Aging is a naturally occurring process inevitably affecting each living human. The brain is adversely affected by aging with increased risks of developing various neurological disorders. Thus, it is essential to investigate practical approaches that can counteract the impact of aging on the brain. Vitamin K2 (Vit. K2) is a naturally occurring vitamin with reported valuable therapeutic effects. The current study highlights the role of Vit. K2 administration in counteracting age-related changes in the brain using naturally aging rats. Three-month-old rats were assigned to two groups: an ageing control group receiving a drug vehicle and an ageing group orally gavaged with Vit. K2 (30 mg/kg, once daily 5 days per week). Treatment was continued for 17 months. Ten three-month-old rats were used as the adult control. Vit. K2 improved functional performance, reduced social anxiety, depressive-like behavior, and enhanced memory performance with concomitant preservation of hippocampal and cerebral cortex tyrosine hydroxylase expression. Biochemically, Vit. K2 administration restored oxidative-anti-oxidative homeostasis in the brain. Vit. K2 modulated inflammatory signaling, as evidenced by suppression in the brain of NLRP3, caspase-1, Il-1β, TNFα, IL-6, and CD68 expression. Concomitantly, histopathological examination revealed consistent hippocampal and cerebral cortex improvement. Thus, it can be inferred that Vit K2 can slow down age-related changes in the brain associated with modulation of NLRP3/caspase-1/Nrf-2 signaling.

## 1. Introduction

Aging is a multifactorial process associated with time-dependent loss of systemic functioning, deterioration of physiological and biochemical functions, disrupted maintenance of tissue homeostasis, and increased vulnerability to disease [1]. It has been recently reported that by 2050, the global number of individuals aged 65 years and more will exceed the number of youths aged 15 to 24 years and will be more than double the number of children below 5 years of age [2].

The aging brain is characterized by loss of volume and neurons, amyloid plaques with α-synuclein accumulation, and neurofibrillary tangles [3]. These changes are more prominent in the hippocampus and the frontal lobes resulting in observable phenotypes related to cognition, attention, memory and learning [4]. Mechanistically, these changes are attributed to mitochondrial changes, increased oxidative stress, compromised intracellular Ca^2+^ homeostasis, impaired DNA repair, inflammation, and neurotrophic factor deficits. Together, all these age-related cellular alterations culminate in impaired functional and structural neuroplasticity [5].

Vit. K is a fat-soluble vitamin, naturally existing in two forms: phylloquinone (Vit. K1) which is synthesized exclusively by leafy green plants, and menaquinone (Vit. K2) which is produced by bacterial and animal tissues and can be found in fermented food and animal products [6]. Both forms of Vit. K have a common menadione (or 2-methyl-1,4-naphthoquinone) nucleus. However, they differ in the number of carbons and double bonds in the 3-polyisoprenoid sidechain [7]. The major forms of Vit. K2, based on the number of prenyl units, are menaquinone-4 (MK-4), MK-7, MK-8, and MK-9 [8].

Endogenously, Vit. K2 is produced either by the conversion of dietary Vit. K1 or by intestinal bacterial activity [9]. In the human colon, numerous bacteria produce Vit. K2, such as bacteroides and enterobacteria, however, the bioavailability of bacterial Vit. K2 is poor, and the diet is the major source of biofunctional available Vit. K2 [10]. Vit. K2 acts as a cofactor essential for optimal body function. Apart from its important role in coagulation, recent reports have linked Vit. K deficiency to the development of cardiovascular diseases, osteoporosis, cancer, and inflammatory and neurodegenerative diseases [11,12,13]. Indeed, various studies have demonstrated Vit K2 to have anti-inflammatory, anti-apoptotic, and anti-oxidant properties, acting as a mitochondrial electron carrier during oxidative respiration [14,15,16].

In the brain, Vit K is a central player in the synthesis and metabolism of sphingolipid which regulates cognitive function via involvement in various neuronal processes, such as proliferation, differentiation, cellular communication, and aging [17,18]. Additionally, Vit K participates in the enzymatic activation of two proteins whose activity contributes to the maintenance of cerebral homeostasis, namely growth-arrest specific 6 protein (Gas-6) which possesses anti-apoptotic, mitogenic and myelinating activity, and protein S which shows neuronal protective efficacy and ability to maintain blood brain barrier integrity [19].

Based on the reported beneficial effects of Vit. K2 on the nervous system, it can be hypothesized that MK-7 may protect against age-related structural and cognitive deterioration in naturally aged rats. Therefore, the current study was designed to evaluate the neuroprotective impact of Vit. K2 in aged rats and to investigate the underlying mechanisms in vivo.

## 2. Materials and Methods

### 2.1. Experimental Procedures

Adult male Sprague–Dawley rats were purchased from the breeding unit of the Holding Company for Biological Products and Vaccines, “VACSERA” (male animals only were used to avoid hormonal fluctuation in females and to produce reproducible data) and kept under standard laboratory conditions for one week acclimatization with free access to chow and water. Thereafter, twenty animals aged 3 months were randomly assigned into two groups (10 rats/group): an **aged vehicle-treated group**, in which the rats received the vehicle (sunflower oil) for 17 months, and an **aged treated group**, in which the rats were orally gavaged with Vit. K2 (Sigma–Aldrich, St. Louis, MO, USA) 30 mg/kg, once daily 5 days per week for 17 months. The selection of doses and the experimental procedure were based on previous and preliminary studies [20,21]. The last group, an additional group, comprising 10 adult rats aged 3 months, was used as an adult control. All experimental procedures were approved by the Research Ethics Committee at the Faculty of Medicine, Mansoura University, Mansoura, Egypt.

At the end of the experimental procedure, animals were sacrificed by thiopental sodium overdose. Blood was withdrawn and centrifuged to prepare the serum for biochemical analyses. The whole brains were immediately dissected, washed with cold-buffered saline 0.9% and divided into two hemispheres. One half/hemisphere (frontal cortex and hippocampus) was appropriately processed to be used for real time polymerase chain reaction (PCR), biochemical analyses of oxidative stress, and ELISA. The other hemisphere (frontal cortex and hippocampus) was fixed in buffered formalin to be used for histopathological and immunohistochemical studies.

### 2.2. Preparation of Tissue Homogenate

The frontal cortex and hippocampus from each rat were homogenized in ice-cold medium of 1.15% KCl (pH 7.4) to give a 10% (*w*/*v*) mixture using a hand-held homogenizer (Omni International, Kennesaw, GA, USA). The supernatants were obtained by centrifugation at 1000× *g* for 15 min and were used for further analyses.

### 2.3. Assessment of Oxidative Stress in Frontal Cortex and Hippocampus Homogenate

Frontal cortex and hippocampus tissue homogenates were used for the measurement of malondialdehye (MDA) as an index of lipid peroxidation, and to assess levels of reduced glutathione (GSH) and superoxide dismutase (SOD) activity as antioxidant markers, using commercially available kits purchased from the Egyptian Company for Biotechnology (SAE) (Obour City, Cairo, Egypt).

### 2.4. Behavioral Tests

#### 2.4.1. Anxiety Test: Crawley’s Sociability Test

The experimental design of this test allows for the evaluation of two aspects of social behavior: social affiliation/motivation and social memory and novelty. The test is based on free choice by a subject rat to spend time in any of three box compartments during experimental sessions that include indirect contact with one or two subjects with which it is unfamiliar. The apparatus for Crawley’s sociability and preference for social novelty test is comprised of a manually constructed acrylic rectangular box divided into three compartments of equal size by retractable doors. Two identical empty round wire cups were placed in the center of each side chambers vertically to contain the stranger rats while the subject rat was placed in the central chamber. The test comprised three sessions (10 min each):

Session I. Habituation: in which the subject rat was placed in the middle chamber with the doors between the chambers closed. Each of the two sides contained an identical empty wire cage.

Session II. Social Affiliation: in which a control stranger rat was placed “Stranger 1” inside a wire containment cup that was located in one of the side chambers. The doors were opened between the compartments to allow free access for the “subject” rat to explore each of the three chambers.

Session III. Social Novelty: in which a second control rat “Stranger 2” was placed inside an identical wire containment cup in the opposite side chamber.

The observations were monitored and recorded by a video camera. After each trial, all chambers were cleaned with 70% ethanol to prevent olfactory cues and bias and to ensure proper disinfection (Supplementary Figure S1). At the end of the experiments, the total time spent by the subject rat in each compartment was calculated [22].

#### 2.4.2. Depressive-like Behavior Testing: Forced Swim Test (FST)

The test is based on placing the rats in a glass cylinder full of water at 25 °C and observing their behavior for 5 min. The immobility time was recorded as a measure of depressive behavior. The rats were placed individually into the swim cylinder. The timer and video recording were set for 5 min. Thereafter, the rats were removed from the water, dried with towels, and returned to their home cages. For each rat immobility time, swimming time, and climbing time were calculated [23].

#### 2.4.3. Memory Testing: Modified T-Maze

The test used a manually run maze T-shaped apparatus. The maze was set so that the central partition was in place and all guillotine doors were raised. The rat was placed in the start area (bottom of the “T”), which was the starting point of each run, and allowed to choose a goal arm. The rat was confined for 30 s in the chosen arm by quietly sliding the door down, and then it was gently removed and returned to the cage for an intertrial interval of 10 min. The rat was replaced in the start area facing away from the goal arms and allowed to choose between the two open goal arms. Each trial took 1–2 min. Alternation was defined as when the rat entered the opposite arm as compared to the previous run. For each rat, one sample trial and five choice runs were performed per day for two days, amounting to a total of 12 trials per rat, and a total of ten possible alternations (Supplementary Figure S2). The percentage of the correct choice (alternation) per animal was calculated as follows: number of correct choice (alternations)/total possible alternations) × 100 [24].

### 2.5. Histopathology and Immunohistochemistry (IHC)

Fixed frontal cortex and hippocampus specimens were dehydrated using ethanol solutions of serial dilutions, cleared in xylene, and embedded in paraffin. The paraffin blocks were sectioned into 4–5 μm, placed on glass slides, and then stained by hematoxylin and eosin (H&E). Slides were examined for hippocampal and cortical changes using built-in camera-light microscopy (OLYMPUS, Tokyo, Japan).

For IHC, 4 μm paraffin sections were mounted, deparafinized and then treated with phosphate buffered saline (PBS) (pH 7.2) for rehydration. The sections were heated for 20 min in citrate buffer (PH 6) for antigen retrieval. Sections were then cooled to room temperature and treated for 10 min with H_2_O_2_ to block the activity of endogenous peroxidase. This was followed by a washing step in PBS. Slides were then incubated with the primary antibodies for CD68 (sc-20060), TNF-α (A0277: ABclonal, Woburn, MA, USA), Nrf-2 (ab89443) and tyrosine hydroxylase (Gene Tex, 113016) at 4 °C overnight; thereafter, slides were washed and then incubated with species-matching secondary antibody. The immune reaction was visualized by applying 3,3′-diaminobenzidine tetrahydrochloride (DAB) for 1 min and slides were counterstained with Mayer’s hematoxylin and then examined by built in camera-light microscopy (OLYMPUS, Tokyo, Japan). Percentage of the immune reaction area was calculated using ImageJ software (NIH, Rockville, MD USA). The measurement units (pixels) produced by the image analyzer program were converted into actual micrometer (μm) units. For each field chosen, the area was masked by a brown binary color to be measured after enclosing the brain tissue inside the standard measuring frame. An objective lens of magnification 100× was used to create an image that covered the entire area to be examined. Six to ten readings were obtained for each specimen according to the colored area and an image analysis program was used for automatic summation of total area. Images were captured at ×400.

### 2.6. Real Time Polymerase Chain Reaction for IL-6 and Nrf-2

Total RNA was extracted from the frontal cortex and hippocampus tissue samples using TRIzol (ThermoFisher Scientific, Waltham, MA, USA). The isolated RNA was tested for quality and quantity using nanodrop. One microgram of extracted RNA was used to synthesize cDNA by reverse transcription using a SensiFAST™ SYBR^®^ Hi-ROX One-Step Kit, catalog no.PI-50217V (Bioline, London, UK). Then, RT-PCR was performed using the StepOne Applied Biosystem, (Foster City, CA, USA) to detect gene expression of IL-6 and Nrf-2 in brain tissue samples using a prepared reaction mix containing 50 ng cDNA, SensiFAST™ SYBR, and the designed primers shown in Table 1. Relative expression of the studied genes was determined using the 2^−ΔΔCt^ formula and glyceraldehyde 3-phosphate dehydrogenase (GAPDH) was used for normalization.

### 2.7. Assessment of Caspase-1, IL-1β and NLRP3 Contents in Frontal Cortex and Hippocampus Homogenate

Protein levels of caspase-1 (BioVision, Milpitas, CA, USA, E4594-100), interleukin-1β (IL-1β), (Cloud-Clone Corporation, Wuhan, China, SEA563Ra) and NLRP3 (Aviva Systems Biology, San Diego, CA 92111, USA, OKCD04232) were assessed in the frontal cortex and hippocampus tissue homogenate using the ELISA technique following the manufacturers’ instructions.

### 2.8. Statistical Analyses

Data were expressed as mean ± SE. For multiple variable comparisons, data were analyzed by one-way analysis of variance (ANOVA). To compare the significance between groups, Tukey’s test was used as a post hoc test. Significance was considered at *p* values less than 0.05. GraphPad Prism 8 (GraphPad Software, San Diego, CA, USA) was used for statistical analyses and graphical presentations.

## 3. Results

### 3.1. Effect of Vit. K2 on Anxiety Testing; Crawley’s Sociability Test

#### 3.1.1. Time (Sec.) Spent in the Empty Chamber, Session I: Habituation

**Figure 1:**
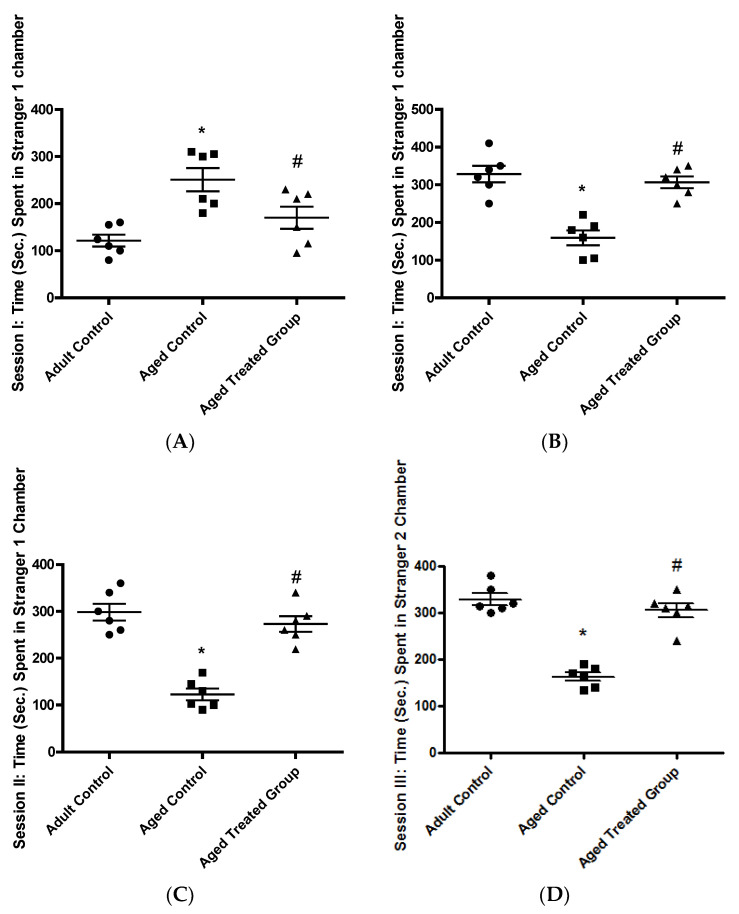
Effect of Vit. K2 administration on social anxiety behavioral test. (**A**) Session I: Time (sec.) spent in the empty chamber, (**B**) Session I: Time (sec.) spent in stranger chamber 1, (**C**) Session II: Time (sec.) spent in stranger chamber 1, and (**D**) Session II: Time (sec.) spent in stranger II chamber. The rats were orally gavaged with viz. K2 (30 mg/kg) once daily 5 days per week for 17 months. Data are expressed as mean ± SE. Statistical analysis was conducted using (ANOVA) followed by Tukey–Kramer’s post hoc test, (*n* = 6, *p* ≤ 0.05). * Significance versus adult control; # significance versus aged control.

As presented in Figure 1A, the mean time spent by the adult control rats in the empty chamber was 121.5 ± 12.8. The aged untreated rats spent a mean time of 250.8 ± 24.6 in the empty chamber which was significantly higher compared to the adult rat control (*p* < 0.01). In 3-month-aged rats treated for 17 months with Vit. K2, the mean time spent in the empty chamber was 170 ± 23.6, which was significantly shorter compared to the aged untreated rats, but without significant difference compared to the adult control rats (*p* < 0.05).

#### 3.1.2. Time (Sec.) Spent in the Stranger 1 Chamber, Session I

In the adult control, the mean time spent with the stranger rat was 328.3 ± 21.8 which was significantly higher compared to the aged untreated control, where the mean time spent with a stranger was 159.2 ± 19.6. For the 3-month-aged rats treated for 17 months with Vit. K2, the mean time spent with stranger I was 306.7 ± 15.4 which was significantly higher compared to the time spent by the aged untreated control (*p* < 0.001), but without significant difference compared to the adult control (Figure 1B).

#### 3.1.3. Time (Sec.) Spent in the Stranger 1 Chamber, Session II: Social Affiliation

In the adult rat control, the mean time spent with stranger I was 289.3 ± 17.9, and this was significantly longer compared to the mean time spent by the untreated aged control (122.7 ± 12.5). Rats on Vit. K2 treatment for 17 months showed improvement as the mean time spent with stranger I was 273.2 ± 16.8 which was significantly longer compared to the time observed with the aged untreated control (*p* < 0.001), but without significant difference in comparison to the adult control, (Figure 1C).

#### 3.1.4. Time (Sec.) Spent in Stranger 2 Chamber, Session III: Social Novelty

In the adult control rats, the mean time spent with stranger 2 was 329.2 ± 12.3, while, for the aged untreated rats the mean time spent with the stranger 2 rat was 163.3 ± 8.9, which was significantly lower compared to the adult control. Rats on Vit. K2 treatment for 17 months spent a longer time with stranger 2 (305.8 ± 14.9), which was significantly longer compared to the aged untreated control, but without significant difference compared to the adult control, (*p* < 0.001), (Figure 1D).

### 3.2. Effect of Vit. K2 on Memory Testing: T-Maze Test

**Figure 2:**
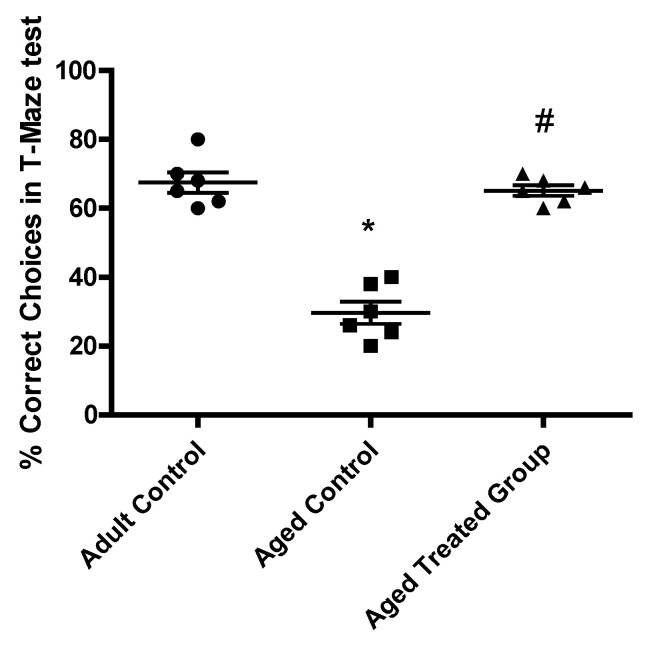
Effect of Vit. K2 administration on memory performance—% correct choices in T-maze test. The rats were orally gavaged with Vit. K2 (30 mg/kg) once daily 5 days per week for 17 months. Data are expressed as mean ± SE. Statistical analysis was conducted using (ANOVA) followed by Tukey–Kramer’s post hoc test (*n* = 6, *p* ≤ 0.05). * Significance versus adult control; # significance versus aged control.

In the adult rat control, the mean % of correct choices in the T-Maze test was found to be 67.5 ± 2.9, while the mean % of correct choices in the aged untreated control was 29.7 ± 3.2, which was significantly lower compared to the adult control. Vit. K2 administration to 3-month-aged rats for 17 months significantly improved the mean % of correct choices of the maze test (65.2 ± 1.5), which was significantly higher compared to the aged untreated control (*p* < 0.001) (Figure 2).

### 3.3. Effect of Vit. K2 on Depressive-like Behavior Testing: Forced Swimming Test

#### 3.3.1. Swimming Time (Sec.)

**Figure 3:**
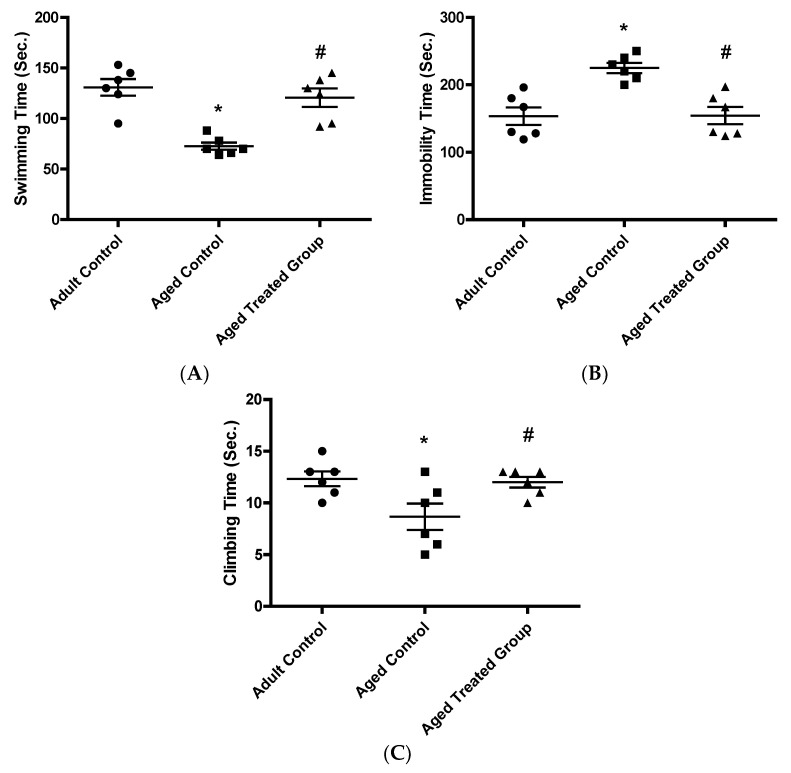
Effect of Vit. K2 administration on depressive-like behavior: forced swimming test (FST). (**A**) Swimming time (sec.), (**B**) Immobility time (sec.), and (**C**) Climbing time (sec.). The rats were orally gavaged with Vit. K2 (30 mg/kg) once daily 5 days per week for 17 months. Data are expressed as mean ± SE. Statistical analysis was conducted using (ANOVA) followed by Tukey–Kramer’s post hoc test, (*n* = 6, *p* ≤ 0.05). * Significance versus adult control; # significance versus aged control.

The adult control rats had a mean swimming time of 130.8 ± 8.3. The aged untreated rats had a mean swimming time of 72.7 ± 3.6, which was significantly shorter compared to the adult control (*p* < 0.001). Treatment of 3-month-aged rats with Vit. K2 for 17 months resulted in a mean swimming time of 120.7 ± 9.1, which was significantly longer compared to the time achieved by the untreated aged control, but without significant difference compared to the adult control (*p* > 0.05) (Figure 3A).

#### 3.3.2. Immobility Time (Sec.)

In context, the adult control scored a mean time for immobility of 153.3 ± 13 and the untreated aged control rats had a mean immobility time of 225 ± 7.6 which was significantly longer in comparison with the adult control (*p* < 0.001). In contrast, rats treated with Vit. K2 for 17 months had a mean immobility time of 154.3 ± 12.7, which was significantly shorter compared to the aged untreated control (*p* < 0.01) (Figure 3B).

#### 3.3.3. Climbing Time (Sec.)

The mean climbing time was 12.3 ± 0.7 in the adult control rats. On the other hand, the climbing time, significantly decreased in the aged untreated rats to 8.7 ± 1.3 (*p* < 0.001). Vit. K2 administration to rats for 17 months significantly restored the mean climbing time to 12 ± 0.5, but without significant difference compared to the adult control (Figure 3C) (*p* < 0.01).

### 3.4. Effect of Vit. K2 on Oxidative (MDA)/Anti-Oxidative (GSH and SOD) Status in Frontal Cortex and Hippocampus

**Figure 4:**
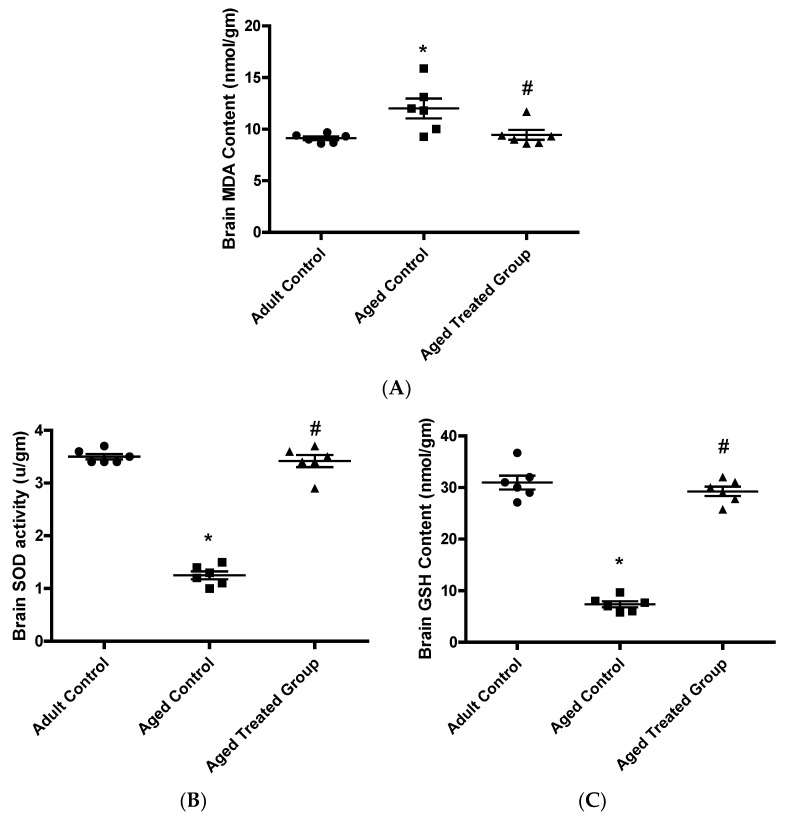
Effect of Vit. K2 administration on frontal cortex and hippocampus oxidative/anti-oxidative biomarkers. (**A**) MDA content, (**B**) SOD activity, and (**C**) GSH concentration. The rats were orally gavaged with Vit. K2 (30 mg/kg) once daily 5 days per week for 17 months. Data are expressed as mean ± SE. Statistical analysis was conducted using (ANOVA) followed by Tukey–Kramer’s post hoc test, (*n* = 6, *p* ≤ 0.05). * Significance versus adult control, # significance versus aged control.

The aged untreated control rats had significantly elevated mean frontal cortex and hippocampus MDA content (12 ± 0.9), and significantly reduced mean frontal cortex and hippocampus GSH concentration (7.4 ± 0.6) and mean SOD activity (1.3 ± 0.1) compared to the adult control. Vit. K2 treatment significantly restored the impaired balance with brain MDA contents significantly reduced to 9.5 ± 0.5 and brain GSH concentrations significantly increased to 29.3 ± 0.9, and SOD activity significantly increased as well (3.3 ± 0.3) compared to the aged untreated rats, (Figure 4).

### 3.5. Effect of Vit. K2 on Frontal Cortex and Hippocampus Gene Expression of IL-6 and Nrf-2

**Figure 5:**
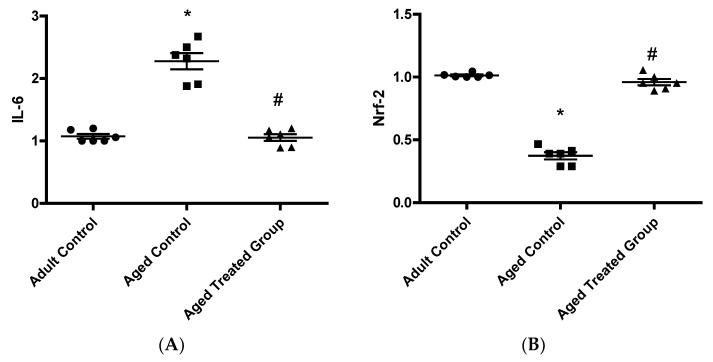
Effect of Vit. K2 administration on frontal cortex and hippocampal relative gene expression of (**A**) IL-6 and (**B**) Nrf-2. The rats were orally gavaged with Vit. K2 (30 mg/kg) once daily 5 days per week for 17 months. Data are expressed as mean ± SE. Statistical analysis was conducted using (ANOVA) followed by Tukey–Kramer’s post hoc test (*n* = 6, *p* ≤ 0.05). * Significance versus adult control; # significance versus aged control.

The aged untreated control rats showed a significant elevation in the relative mean gene expression of IL-6 (2.32 ± 0.22), with a significant parallel suppression in the mean gene expression of Nrf-2 (0.39 ± 0.05), compared to the adult control. On the other hand, Vit. K2 treatment induced significant suppression in the relative genetic expression of IL-6 (1.06 ± 0.09), with a parallel restoration in the genetic expression of frontal cortex and hippocampal Nrf-2 (0.95 ± 0.05) compared to the aged untreated control (Figure 5).

### 3.6. Effect of Vit. K2 on Caspase-1, Il-1β, and NLRP3 Contents in Frontal Cortex and Hippocampus

**Figure 6:**
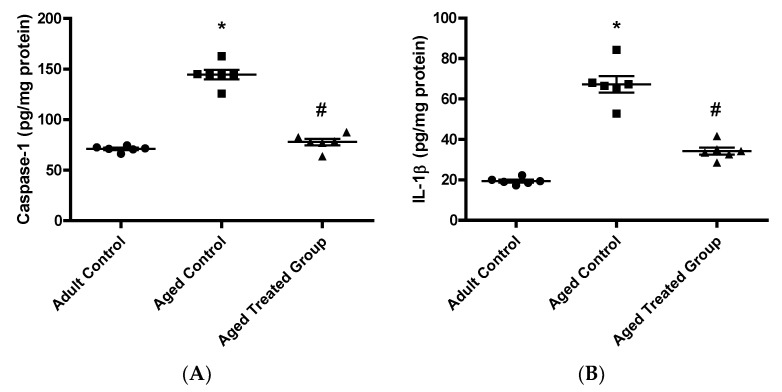

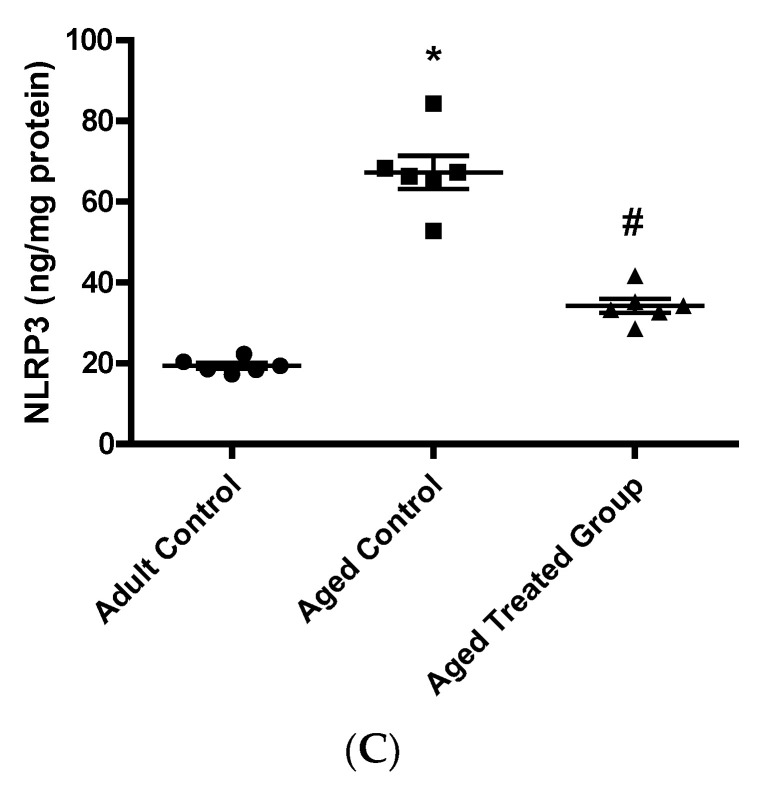
Effect of Vit. K2 administration on frontal cortex and hippocampus content of: (**A**) caspase-1, (**B**) IL-1β1 and (**C**) NLRP3. The rats were orally gavaged with Vit. K2 (30 mg/kg) once daily 5 days per week for 17 months. Data are expressed as mean ± SE. Statistical analysis was conducted using (ANOVA) followed by Tukey–Kramer’s post hoc test, (*n* = 6, *p* ≤ 0.05). * Significance versus adult control; # significance versus aged control.

In context with the previous behavioral and biochemical changes, aged untreated control rats had a significant elevation in brain hippocampal contents of caspase-1 (144.5 ± 10.66), IL-1β (67.27 ± 1.51), and NLRP3 (2.28 ± 0.19) compared to the adult control. Rats treated with Vit. K2 for 17 months showed a significant reduction in brain hippocampal caspase-1 content (77.97 ± 7.235), IL-1β (34.23 ± 3.87), and NLRP3 (1.26 ± 0.05) compared to the aged untreated control rats (Figure 6).

### 3.7. Effect of Vit. K2 on the Histopathological Changes in the Hippocampus and the Frontal Cortex of Naturally Aging Rats

**Figure 7:**
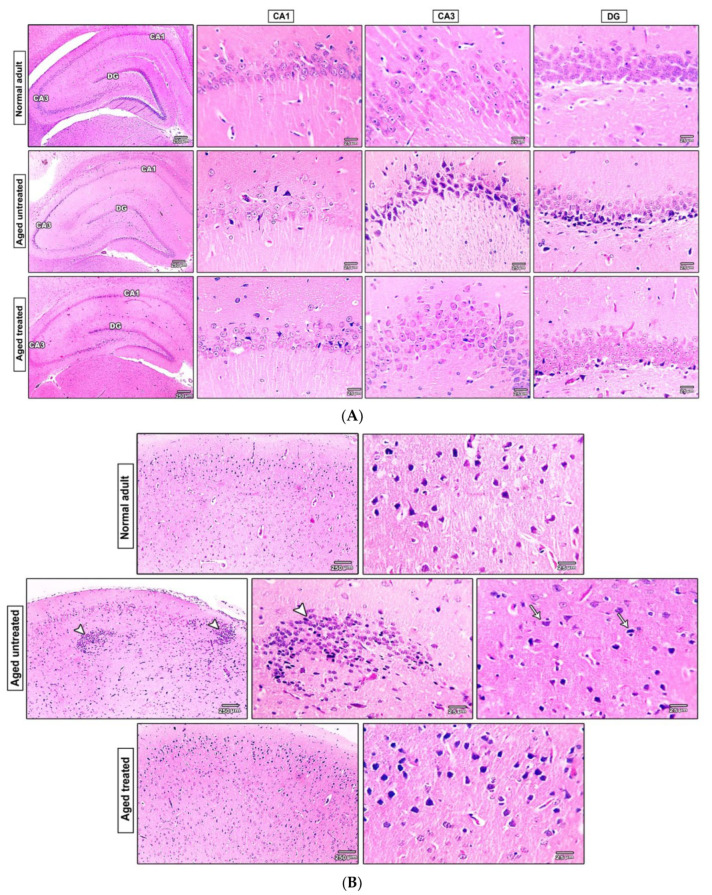
(**A**) Microscopic pictures of HE-stained hippocampal sections showing normal neurons in 3 examined regions, CA1, CA3, and DG, in adult control. Hippocampal sections from the untreated aged group showed degenerated neurons exhibiting acidophilic cytoplasm and dark nuclei (arrows) in 3 examined regions but most prominent in CA3 and DG. Hippocampal sections from Vit. K2-treated rats for 17 months showing few degenerated neurons (arrows) in CA1 and DG with improved histological picture of CA3. Magnifications ×40 bar 200 and ×400 bar 25. (**B**) Microscopic pictures of HE-stained cerebral cortical sections showing normal pyramidal cells and glial cells in the adult group. Cerebral cortical sections from the adult control group showing multifocal areas of gliosis around degenerated neurons (arrowheads) with satellitosis (arrows). Cerebral cortical sections from the treated aged group showed normal histology of pyramidal cells and glial cells, ×400 bar 25.

The microscopic pictures of H&E-stained hippocampal sections revealed normal neurons in three examined regions, CA1, CA3, and DG, in the adult control. Hippocampal sections from the untreated aged control group revealed degenerated neurons exhibiting acidophilic cytoplasm and dark nuclei (arrows) in three examined regions but most prominently in CA3 and DG. Hippocampal sections from the treated aged group revealed retraction of histopathological features of aging with only few degenerated neurons (arrows) in both CA1 and DG, and an improved histological picture of CA3 (Figure 7A).

The cerebral cortical sections from the adult control revealed normally arranged pyramidal cells and glial cells. The cerebral cortical sections from the adult control revealed multifocal areas of gliosis around degenerated neurons (arrowheads) with satellitosis (arrows). Cerebral cortical sections from rats treated with Vit. K2 for 17 months revealed a significant recovery with restoration of the normal histology of pyramidal and glial cells (Figure 7B).

### 3.8. Effect of Vit. K2 on CD68 Expression in Frontal Cortex and Hippocampus

**Figure 8:**
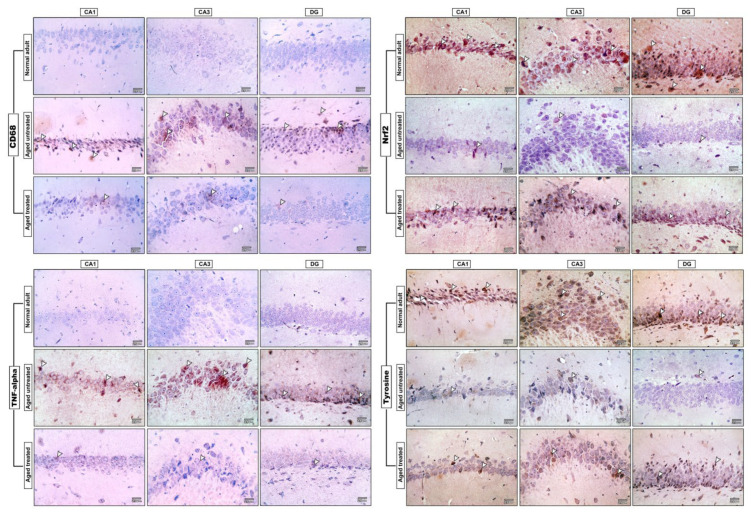
Microscopic pictures of immuno-stained hippocampal, CA1, CA3 and DG sections against CD68, TNF-alpha, Nrf2 and tyrosine hydroxylase. IHC counterstained with Mayer’s hematoxylin. Arrow heads point to positively stained neurons (×400 bar 25).

**Figure 9:**
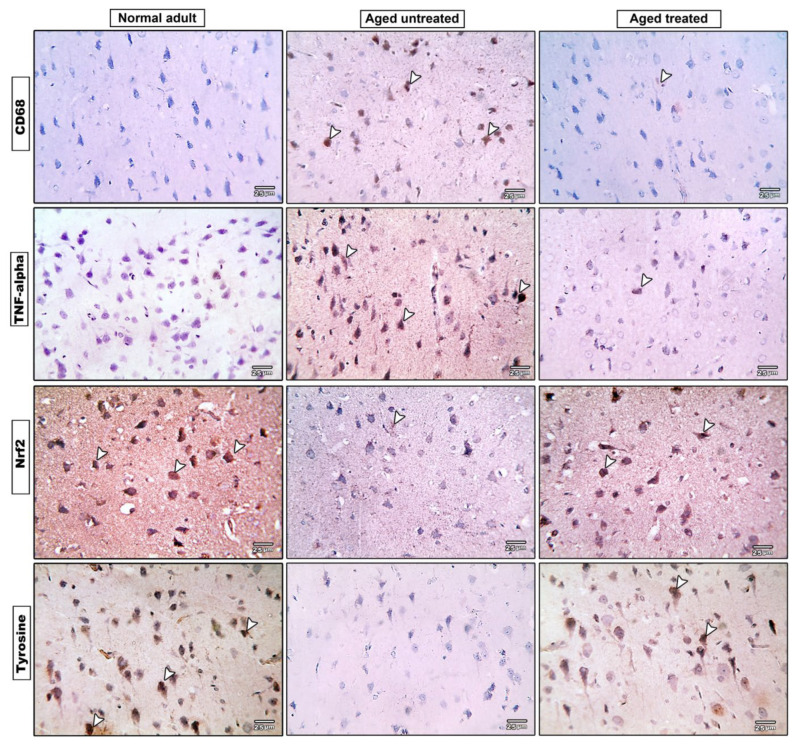
Microscopic pictures of immuno-stained cerebral cortical (CC) sections against CD68, TNF-alpha, Nrf2 and tyrosine hydroxylase. IHC counterstained with Mayer’s hematoxylin. Arrow heads point to positively stained neurons (×400 bar 25).

**Figure 10:**
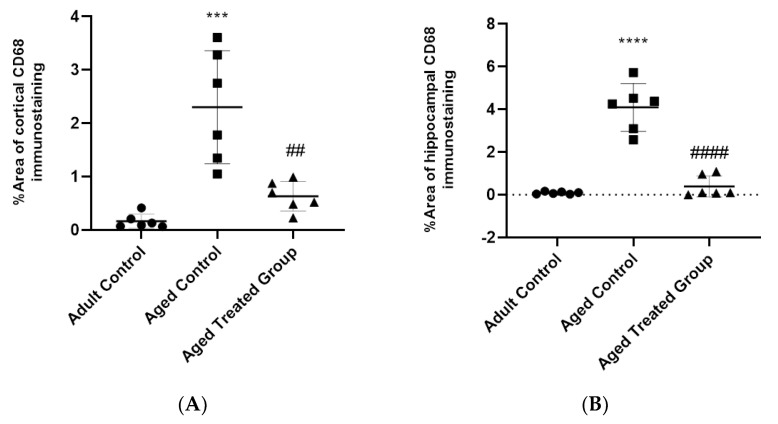

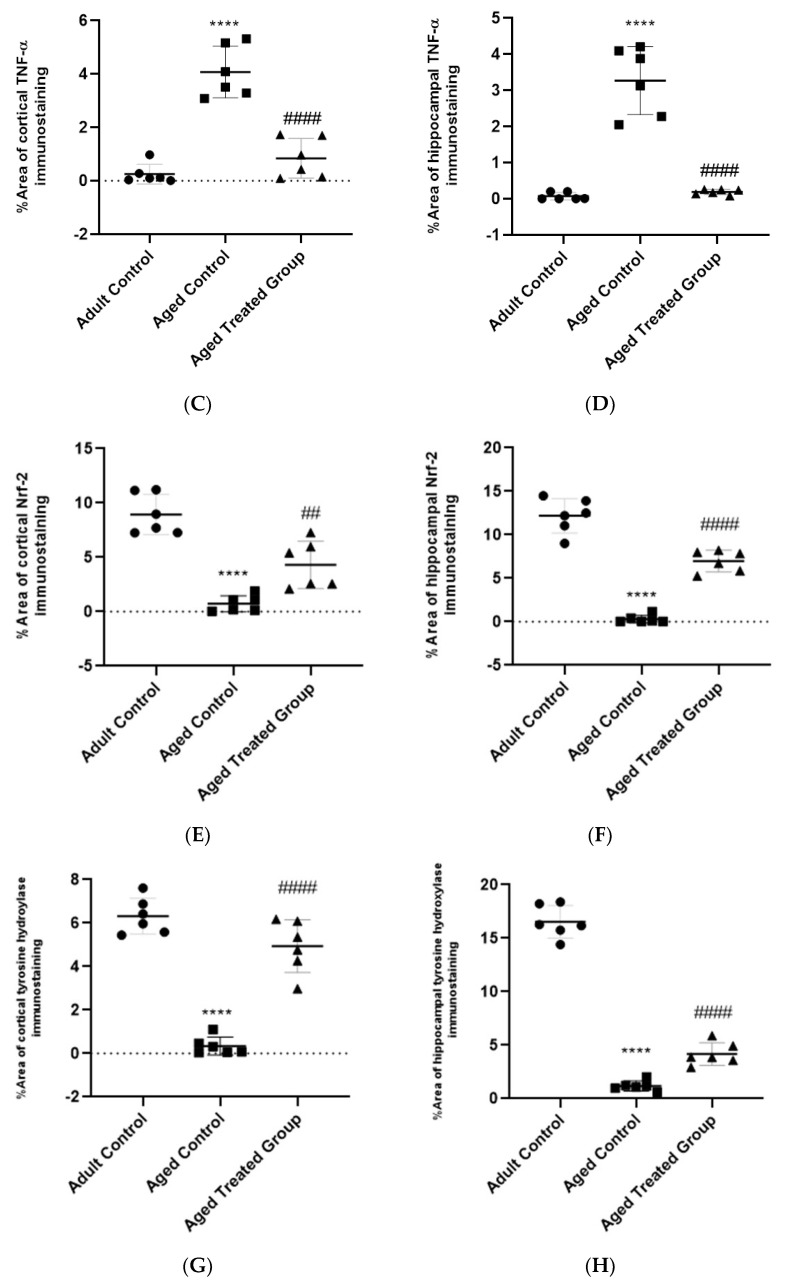
% area of immuno-staining cortical and hippocampal CD68 (**A**,**B**), TNF-alpha (**C**,**D**), Nrf2 (**E**,**F**) and tyrosine hydroxylase (**G**,**H**). Data are expressed as mean ± SE. Statistical analysis was conducted using (ANOVA) followed by Tukey–Kramer’s post hoc test, (*n* = 6, *p* ≤ 0.05). *** Significance versus adult control at *p* ˂ 0.001; **** Significance versus adult control at *p* ˂ 0.0001; ## significance versus aged control at *p* ˂ 0.01, #### at *p* ˂ 0.0001.

Microscopic pictures of the immuno-stained hippocampal (CA1, CA3, and DG) (Figure 8) and cerebral (Figure 9) cortex sections against CD68 revealed negative expression in the adult control group, and prominent positive brown expression in the neurons of the untreated aging control group. CD68 expression significantly increased by 45- and 12-fold, respectively, in the hippocampus and cerebral cortex compared to the adult control. Vit. K2-treated rats, on the other hand, revealed only mild positive brown expression against CD68 in both CA1 and CA3; meanwhile, the reaction returned negative values in CC and DG. Cd68 expression significantly declined by 90% and 69% in the hippocampus and the cerebral cortex of Vit. K2-treated aging rats compared to the aging control (Figure 10A,B).

### 3.9. Effect of Vit. K2 on TNF-α Expression in Frontal Cortex and Hippocampus

Microscopic pictures of the immuno-stained hippocampal (CA1, CA3, and DG) (Figure 8) and cerebral cortex (Figure 9) sections against TNF-α revealed negative expression in the adult control group. On the other hand, prominent positive brown expression was detected in the neurons in the aging untreated control group. The positive brown expression against TNF-α markedly declined in the examined sections from 3-month-aged rats treated with Vit. K2 for 17 months compared to the untreated control group. Quantitatively, aging was associated with a significant increase in TNF-α expression in the hippocampus and the cerebral cortex by 82- and 16-fold, respectively, compared to the adult control. On the other hand, Vit K2 treatment significantly suppressed hippocampal and cerebral cortex expression of TNF-α by 95% and 80%, respectively, compared to the aging control group (Figure 10C,D).

### 3.10. Effect of Vit. K2 on Nrf-2 Expression in Frontal Cortex and Hippocampus

Microscopic sections of immuno-stained hippocampal (CA1, CA3, and DG) (Figure 8) and cerebral cortex (Figure 9) sections against Nrf-2 revealed strong positive brown expression in the neurons in the adult control group. Nrf-2 expression significantly declined by 98% and 92% in the hippocampal and cerebral cortex sections compared to the adult control. Mild positive brown expression in the neurons in the untreated aging control group compared to the adult control group was evident. On the other hand, moderate positive brown expression in the neurons was observed in rats treated with Vit. K2 for 17 months. Vit K2 induced a significant 23-fold and 6-fold increase in hippocampal and cerebral cortex expression of Nrf-2, respectively, compared to the aging control group (Figure 10E,F).

### 3.11. Effect of Vit. K2 on Tyrosine Hydroxylase Expression in Frontal Cortex and Hippocampus

Microscopic pictures of the immuno-stained hippocampal (CA1, CA3 and DG) (Figure 8) and cerebral cortex (Figure 9) sections against tyrosine hydroxylase in the adult control group revealed marked positive brown expression in the neurons; very mild positive brown expression in the neurons was detected in the untreated aging control group. Tyrosine hydroxylase expression significantly declined by 93% and 95% in the hippocampal and cerebral cortex sections, respectively, compared to the adult control group. Vit. K2 treatment for 17 months, on the other hand, was associated with only moderate positive brown expression in the neurons in the aging treated group. Tyrosine hydroxylase expression significantly increased by about 3.6- and 14-fold in the hippocampal and cerebral cortex sections compared to the aging untreated control (Figure 10G,H).

## 4. Discussion

Aging is a normal physiological process. For humans, it is usually associated with negative consequences that impair the quality of life of the aging individual. Additionally, aging imposes huge burdens on society, health care systems and the economy. Indeed, neurodegenerative disorders and deteriorated cerebral performance are considered serious disabling consequences of aging. Given the progressive aging of the global population, data indicate that cases of dementia will double between 2020 and 2040, rising to 81 million, consequently burdening society and health systems [19].

Thus, it has become essential to investigate novel approaches with beneficial and applicable impacts to improve the central nervous functions in aging individuals and to minimize the negative consequences of the aging process.

The central nervous system (CNS) consumes 20% of the body’s oxygen and is highly susceptible to oxidative damage. Specifically, neurons are post-mitotic non-dividing cells, making them highly sensitive to oxidative stress [25]. Despite the various theories suggested to explain the pathogenic mechanisms of aging, free radical theory, later termed the oxidative stress theory of aging, is most relevant [26]. The theory proposes that increased reactive oxygen species (ROS) formation is an important generator of age-related cellular lesions and disturbances, leading eventually to the diminution of cerebral activities [27]. Nevertheless, supplementation with antioxidants has been reported to slow the aging process [28].

Recently, Vit. K2 status has been strongly correlated with brain health [25]. Various in vitro and in vivo studies have highlighted the role of Vit. K in brain cell development and survival. In this regard, a direct correlation between low Vit. K dietary intake, or its serum concentration, and deteriorated cognitive and behavioral performances has been reported in a population 65 years and older [29]. Moreover, research findings confirmed Vit. K2 to protect neural cells against anti-beta amyloid antibodies toxicity [25].

Vit. K2 has been reported to be involved in the synthesis of sphingolipids, an important class of lipids that are present in high concentrations in brain cell membranes and has been demonstrated to participate in crucial cellular events, including signaling, and the proliferation, differentiation, senescence, transformation and survival of brain cells. Moreover, in recent years, studies have linked alterations in sphingolipid metabolism to age-related cognitive decline and neurodegenerative disorders [30]. Indeed, Vit. K deficiency has been confirmed in aged rats with cognitive deficits [31]. Moreover, Alzheimer’s disease patients were reported to have reduced serum Vit. K2 levels [19]. Furthermore, aged individuals on anticoagulant therapy, which primarily functions by antagonizing Vit. K, have been reported to display cognitive impairment [32].

The current study presents evidence on the positive impact of administering Vit. K2 to rats, naturally aged for 17 months, on functional, behavioral, biochemical, and histopathological measurement scales. The aging rats demonstrated a deterioration in behavioral performance with concomitant impairment in oxidative/anti-oxidative balance in the brain, increased inflammatory genetic expression, signaling, and retraction in cerebral content of tyrosine hydroxylase, the enzyme responsible for the conversion of tyrosine into dopamine—an important neurotransmitter in the brain.

The aging brain is characterized by various pathological and physiological changes in memory and cognitive functions associated with anxiety, mood disruption, and depression [33]. An animal model of human behavior represents a complex of cognitive and/or emotional processes, which are translated from animals to humans. Tests commonly used in novel drug discovery focus especially on anxiety, depression-like behaviors, and memory [34]. The current study investigated all three aspects. Anxiety was assessed using Crawley’s sociability test, a forced swimming test was used to assess depressive-like behavior, and a T-maze test was used to assess memory performance. The current results confirmed the incidence of anxiety, depressive-like behaviors, and memory deterioration in the aged untreated rats. However, these behavioral changes were attenuated by Vit. K2 administration.

Altered dopamine synthesis and signaling, with a concomitant profound impact on cognitive and memory functions, is a hallmark of the aging brain [35,36]. Tyrosine is the precursor of brain catecholamines. It is converted to dopamine via L-dopa and the enzymes tyrosine hydroxylase and aromatic l-amino acid decarboxylase. Interestingly, tyrosine hydroxylase has been reported to significantly decrease during the aging period in rats. Moreover, tyrosine hydroxylase has also been reported to be substantially inactivated by oxidation under increased oxidative load in the aging brain [37], which gives credence to the results of the current study.

The aging process, as observed in the current study, was associated with a significant increase in frontal cortex and hippocampus content of MDA and retraction in GSH concentration and SOD activity as markers for enhanced ROS generation. In context, both gene and protein content of NRf-2, a cellular sensor for oxidative status, were significantly decreased. As previously mentioned, oxidative stress theory concerning aging and enhanced oxidative status has significantly contributed to understanding of the inactivation of the enzyme tyrosine hydroxylase and, hence, deterioration in behavioral performance. Interestingly, Vit. K2 administration to rats naturally aged for 17 months, in the current study, significantly attenuated oxidative load in the brain, which may have contributed to the observed preservation of brain tyrosine hydroxylase contents and, hence, the observed functional and behavioral improvement in the social anxiety and depressive-like behavioral testing, as well as memory performance.

Many types of neuroinflammatory and neuroimmune pathways have been implicated in brain aging. Within the aging brain, an increase in macrophage infiltration, microglia priming, and/or microglia activation have been observed. The chronic activation of microglia in the aging brain, particularly within the hippocampus, is thought to cause persistent neuroinflammation, detrimentally affecting cognitive function [38]. When the microglia are activated, they initiate an inflammatory cascade, releasing an excess of proinflammatory mediators including TNF-α, IL-1β, and IL-6 [39].

In the current study, parallel to the impaired oxidative status, the aging brain revealed significant inflammatory changes, as confirmed by increased gene expression of IL-6 and the brain’s content of TNF-α. Meanwhile, the hippocampus and the cerebral cortex of the aging rats showed significant elevation of CD68 expression, implying macrophage infiltration. On the other hand, Vit. K2 administration induced a significant improvement in the brain’s biochemical and histopathological inflammatory status.

Age-related inflammation is characterized by a chronic mild inflammation called “inflammaging”, which negatively impacts CNS functions. NLRP3 inflammasome activation is linked to inflammation-induced cognitive function decline and neuropathological changes with aging. Notably, the NLRP3 inflammasome can be activated by ROS and, thus, aging-associated NLRP3 activation makes the aged brain vulnerable to cognitive dysfunction [40]. NLRP3 activation induces the cleavage and the release of mature forms of IL-1β through the activation of inflammatory caspases [41]. Caspase-1 is activated via proximity-induced autocatalytic activation upon recruitment to an inflammasome and active caspase-1 cleaves the cytokine pro-IL-1β into its mature and biologically active form [42].

In the current study, the frontal cortex and hippocampus showed a significant increase in content of caspase-1, IL-1β and NLRP3 in the aged group, highlighting the role of NLRP3 activation in aging and deterioration of cognitive functions. On the other hand, Vit. K2 administration induced a significant improvement in frontal cortex and hippocampal contents of caspase-1, IL-1β, and NLRP3, suggesting the crucial role of Vit K2-induced modulation of caspase-1/IL-1β/NLRP3 signaling in attenuation of age-related changes in the brain’s status and cognitive functions.

Vit. K has been reported to demonstrate anti-apoptotic and anti-inflammatory impacts mediated by the activation of both Gas-6, which demonstrated anti-apoptotic, mitogenic, and myelinating activity, and protein S, which offered neuronal protection during ischemic/hypoxic injury, both in vivo and in vitro [9]. Murine peritoneal macrophages treated with Gas6 demonstrated decreased caspase-1 activation and IL-1β release upon stimulation with ATP, a classic NLRP3 inflammasome activator. Mechanistically, Gas-6 stimulates autophagy, a well-known intracellular event that prevents mitochondrial dysfunction by reducing the release of endogenous inflammasome agonists, such as ROS and oxidized mitochondrial DNA [43]. Gas-6 also reduced the release of IL-1β by THP-1 cells after incubation with silica, another well-known NLRP3 inflammasome activator [44]. The transcription of inducible antioxidant genes is chiefly controlled by Nrf2 [45]. Once the oxidative balance is impaired, inflammatory response and mitochondria-related cell death follow [46].

Thus, in light of the reported results relating to signaling pathways and the observed results, it can be inferred that Vit. K2 administration attenuated aging-related changes by modulation of NLRP3/caspase-1/Nrf-2 signaling; inflammatory/anti-oxidative signaling. Vit K2 suppressed NLRP3 activation, enhanced Nrf-2 expression, inhibited caspase-1-mediated activation of IL-1β1, with subsequent suppression of inflammatory changes in the frontal cortex and hippocampus, as confirmed by decreased gene expression of IL-6, and hippocampal and cerebral cortex expression of TNF-α. Meanwhile, Vit. K2 suppressed hippocampal and cerebral cortex CD68 expression, implying decreased macrophage infiltration with decreased ROS generation and inflammasome activation.

## 5. Conclusions

Vit. K2 demonstrated very promising impacts in hindering aging-related behavioral, functional, biochemical, and histopathological changes in aged rats. This was underlined by a modulatory impact on NLRP3/caspase-1/Nrf-2 signaling with preservation of frontal cortex and hippocampal tyrosine hydroxylase content—a major contributor to the preservation of cognitive functions. Vit. K2 is proposed as a promising approach to attenuate age-related disorders and to preserve cognitive functions in aging individuals.

## Figures and Tables

**Table 1:** Primer sequence of target genes.

Gene Symbol	Primer Sequence from 5′-3′	Gene Bank Accession Number
***β-actin***	F: TCCGTCGCCGGTCCACACCC R: TCACCAACTGGGACGATATG	NM_031144.3
***Nrf2***	F: AGCAGGACATGGATTTGATT R: CTTCTCCTGTTCCTTCTGGA	XM_032903520.1
***Il-6***	F: TCCTACCCCAACTTCCAATGCTC R: TTGGATGGTCTTGGTCCTTAGCC	M26745

## Data Availability

Data is contained within the article.

## Supplementary Materials

The following supporting information can be downloaded at: https://www.mdpi.com/article/10.3390/antiox11030514/s1, Supplementary Figure S1: Apparatus for anxiety testing; Crawley’s sociability test. Supplementary Figure S2: Apparatus for memory testing; Modified T-maze.
