# Supplementary material for: Vitamin K2 (Menaquinone-7) Reverses Age-Related Structural and Cognitive Deterioration in Naturally Aging Rats
Source: Antioxidants (Basel). 2022 Mar 8;11(3):514. doi: 10.3390/antiox11030514 (PMC8944720; doi:10.3390/antiox11030514)
Supplement: Supplementary file 1 [file antioxidants-11-00514-s001.zip › antioxidants-1578988-spplementary materials revised.pdf]

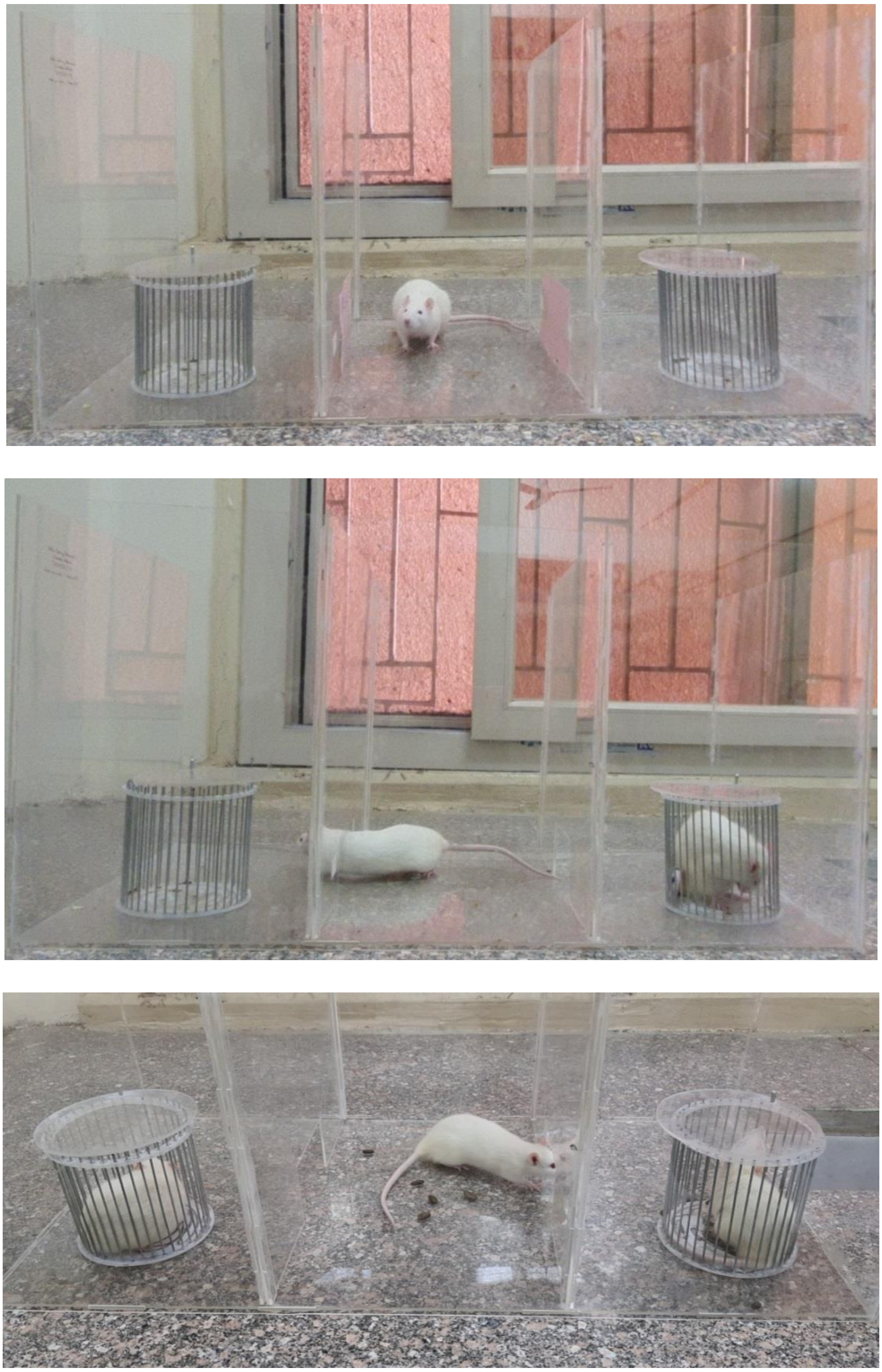

A

B

C

Supplementary Figure S1: apparatus for anxiety testing; Crawley's sociability test. A: session I, B: Session II, C: Session III.

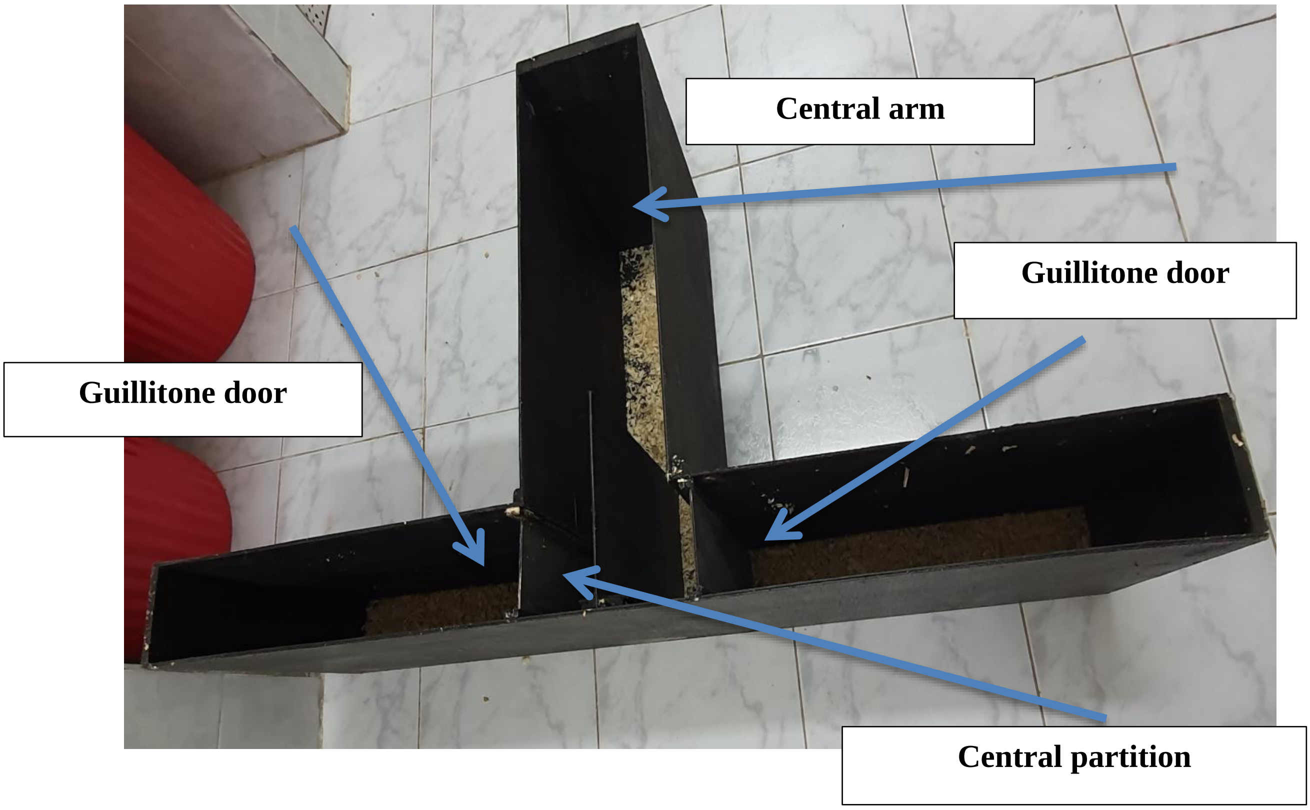

Supplementary Figure S2: apparatus for Memory testing; Modified T-maze
